# Rebuilding Biodiversity of Patagonian Marine Molluscs after the End-Cretaceous Mass Extinction

**DOI:** 10.1371/journal.pone.0102629

**Published:** 2014-07-16

**Authors:** Martin Aberhan, Wolfgang Kiessling

**Affiliations:** 1 Museum für Naturkunde, Leibniz Institute for Evolution and Biodiversity Science, Berlin, Germany; 2 GeoZentrum Nordbayern, Paläoumwelt, Universität Erlangen−Nürnberg, Erlangen, Germany; Australian Museum, Australia

## Abstract

We analysed field-collected quantitative data of benthic marine molluscs across the Cretaceous–Palaeogene boundary in Patagonia to identify patterns and processes of biodiversity reconstruction after the end-Cretaceous mass extinction. We contrast diversity dynamics from nearshore environments with those from offshore environments. In both settings, Early Palaeogene (Danian) assemblages are strongly dominated by surviving lineages, many of which changed their relative abundance from being rare before the extinction event to becoming the new dominant forms. Only a few of the species in the Danian assemblages were newly evolved. In offshore environments, however, two newly evolved Danian bivalve species attained ecological dominance by replacing two ecologically equivalent species that disappeared at the end of the Cretaceous. In both settings, the total number of Danian genera at a locality remained below the total number of late Cretaceous (Maastrichtian) genera at that locality. We suggest that biotic interactions, in particular incumbency effects, suppressed post-extinction diversity and prevented the compensation of diversity loss by originating and invading taxa. Contrary to the total number of genera at localities, diversity at the level of individual fossiliferous horizons before and after the boundary is indistinguishable in offshore environments. This indicates an evolutionary rapid rebound to pre-extinction values within less than *ca* 0.5 million years. In nearshore environments, by contrast, diversity of fossiliferous horizons was reduced in the Danian, and this lowered diversity lasted for the entire studied post-extinction interval. In this heterogeneous environment, low connectivity among populations may have retarded the recolonisation of nearshore habitats by survivors.

## Introduction

The study of mass extinction events can reveal patterns of selective survival and the dynamics of ecosystem recovery over evolutionary time scales [1]–[3]. Apart from developing models of the processes involved in recovery dynamics [2], [4], such information can be gleaned from empirical studies of the geological past.

The mass extinction at the Cretaceous-Palaeogene (K-Pg) boundary is among one of the best studied Phanerozoic bioevents [5]. Geographically, the scope of studies encompasses local, regional, and global scales [6]–[8]. Yet, our understanding is still limited about how global signals of diversity dynamics arose as a result of the accumulation of events at local and regional scales. A general challenge is the reciprocal relationship between the geographical extent of a study and its temporal resolution. The 11-myr binning resolution of global occurrence compilations of marine macrofauna [8] is too coarse to uncover the dynamics of post-extinction diversity rebounds, whereas local-scale analyses may lack general validity.

Here we construct high-resolution diversity trajectories from benthic marine molluscs at two fossiliferous K-Pg boundary sites in Patagonia and incorporate information on the evolutionary and biogeographical characteristics of the fauna and on species abundances. The two studied sites represent marginal to shallow marine (nearshore) and middle shelf environments (offshore). We focus on evolutionary patterns and longer-term diversity trends across the K-Pg interval. In particular, we test two null hypotheses: Within the first *ca* 4 million years (myr) after the extinction event (1) the diversity of mollusc assemblages is indistinguishable from that of pre-extinction faunas; (2) the transient loss of diversity through global extinctions and local extirpations was compensated for by gains via immigrating and newly originating taxa. We demonstrate that hypothesis (1) is only partly supported, and hypothesis (2) can be rejected by empirical evidence.

## Materials and Methods

No permits were required for the described study, which complied with all relevant regulations.

### Sampling localities

We analysed two stratigraphic sections in the Argentine part of Patagonia. The first one is a composite section from the adjacent localities Bajada del Jagüel (BJG, 38°6′S, 68°23′W) and Opaso (38°8′S, 68°24′W), located in the Neuquén Basin north of the city of Neuquén. The second section, close to Estancia San Ramón in the middle Chubut River valley (42°41′S, 69°51′W), is located in the Cañadón Asfalto Basin. During latest Cretaceous–earliest Palaeocene times the sea in both areas formed narrow embayments, which opened to the Atlantic Ocean to the east [9].

The sections at BJG and Opaso consist of monotonous, middle shelf siliciclastic mudstones of the Jagüel Formation, representing open marine sedimentation below, or close to, the storm wave base [10]. In the following they are referred to as ‘offshore’. Because the section at Opaso virtually lacks a Maastrichtian record to which the Danian samples could be compared, the faunal data from BJG and Opaso were arranged chronologically according to their distance from the K-Pg boundary and integrated into a single succession (i.e. our offshore section). We thereby assumed equal sedimentation rates at these close-by localities. Faunal samples were derived from 11.4 metres below the K-Pg boundary to 27.5 metres above the boundary.

In San Ramón, faunal sampling extends over 188 m of varied sedimentary rocks of the Lefipán Formation. The Maastrichtian to early Palaeocene succession comprises siliciclastic mudstones, siltstones, sandstones, and conglomerates. Litho- and biofacies indicate deposition in a marginal to shallow marine, tide-dominated deltaic setting with tidal bars, prodelta, and delta plain environments, which are represented in both the Maastrichtian and the Danian [9]. In this paper, the aforementioned deltaic setting is referred to as ‘nearshore’. Salinity was an important factor affecting the composition and ecological structure of the fauna in these environments. The uppermost Maastrichtian is characterized by virtually monospecific assemblages of the bivalve genus *Corbicula* owing to oligohaline to mesohaline salinity, whereas more fully marine conditions existed below and above this interval [9]. These very low-diversity brackish-water assemblages were excluded from our analyses because they represent a different environment.

### Biostratigraphy and interval lengths

In BJG, the K-Pg boundary is at the erosive base of a coarse-grained, tuffaceous sandstone bed [10], [11]. Calcareous nannofossils prove the presence of the first Cenozoic nannofossil zone (NP1 or NNTp1) in the upper part of the sandstone layer, and younger subzones in the mudstones overlying the sandstone [10]. The mudstones below the sandstone are late Maastrichtian in age [10], [11]. According to Keller et al. [11], the 11.4 metres of section below the K-Pg boundary, which yielded the late Maastrichtian quantitative samples in our study, are characteristic of the nannofossil zone CC26 and the *A. mayaroensis* and *P. hariaensis* foraminiferal zones (zonal names according to [12]). In Opaso, the K-Pg boundary sandstone is exposed about half a metre above the base of the sampled section. Micro- and nannofossils indicate the presence of early (NP1-NP3?/P1a-b) to late Danian (NP4/P1c) biozones [13]. Applying the ages given in Gradstein et al. [12], the samples of BJG and Opaso extend from ∼2 myr before the K-Pg boundary to ∼4 myr after the boundary. Because the earliest Danian planktonic foraminiferal zones P0 and Pα, the latest Maastrichtian *P. hantkeninoides* foraminiferal zone, and impact tracers are missing, the sections at BJG and Opaso are stratigraphically incomplete [10], with possibly up to 500 kyr of the earliest Danian and 300 kyr of the latest Maastrichtian missing [10], [11]. In summary, the amount of time represented by the Danian strata is about twice that of the Maastrichtian strata.

In the nearshore San Ramón section, a distinct boundary layer is not preserved. However, the presence of latest Maastrichtian and earliest Danian dinoflagellate markers and age-diagnostic palynomorphs indicate a very minor hiatus [14]. A more detailed biostratigraphical subdivision is not available in this very shallow-water setting. Because depositional environments are the same below and above the boundary, and because the sampled Maastrichtian succession (100 metres) is similar in thickness to that of the Danian (88 metres), we tentatively assume roughly equal time spans for the studied pre- and post-extinction intervals.

### Analytical methods

We quantitatively sampled 157 single beds of uniform lithology for macroinvertebrates, comprising a total of 5,547 individuals. Bivalves and gastropods dominate the macrofauna. Taxa were identified as precisely as possible, mostly at species level. Our analyses focus on genus diversity of bivalves and gastropods. We elevated subgenera to genus rank and omitted all taxa not resolvable at least to the genus level. Because most genera are represented by a single species at the studied localities, the number of taxa is almost identical at genus and species level. Following standard procedures [15] the number of individuals of bivalves was determined as the sum of articulated specimens plus the larger number of right or left valves of disarticulated specimens plus one-half the number of fragments which could not be assigned to either right or left valves but make up more than 50% of a valve. In one special case – *Entolium membranaceum,* an equivalved and equilateral pectinoid with symmetrical auricles – distinction of the right and left valves was impossible, thus numbers of specimens were halved to yield numbers of individuals. Gastropods were counted when more than 50% of the shell was preserved.

To avoid large variations in sample size, we pooled samples of successive fossiliferous beds until at least 100 individuals were reached or until the lithology changed markedly. In the offshore section, this resulted in 24 pooled samples with a mean size of 129.1±7.1 (standard error) individuals. The 28 pooled nearshore samples had a mean of 63.1±5.5 individuals.

We focus on two measures of diversity. The first one determines the standardised number of genera for each of the pooled samples. We refer to this measure as sample-level diversity [16]. We standardised sample-level diversity using the ‘shareholder quorum’ subsampling method developed by Alroy [8], [17]. This method involves random sampling of individuals to equal frequency coverage, the shareholder quorum. In our analyses the quorum was set to 0.8 and our values are averages of 100 iterations. The shareholder quorum is superior to traditional subsampling methods (e.g., rarefaction) in that variations in evenness are explicitly taken into account. Therefore, it is less biased by different abundance distributions and represents a nearly unbiased estimate of richness.

Our second measure of diversity aims at comparing the total number of Danian genera in a section with that of Maastrichtian genera in the same section. This measure is termed total (Maastrichtian or Danian) diversity. To this end, the genera of the pooled and standardised samples were summed up over consecutively younger samples for the Maastrichtian and for the Danian part of the section, respectively. Genera are added to the count of total diversity at the sampling level at which they first appeared in the succession and are considered to persist up to the K-Pg boundary (for Maastrichtian total diversity) or to the youngest Danian sample (for Danian total diversity). This procedure constructs sample-based accumulation curves [18], whereby the sequence of samples is ordered in time via stratigraphy. This approach assumes that diversity accumulates during both the Maastrichtian and the Danian. While confidence intervals on stratigraphic ranges of genera support this approach, we emphasize that this procedure is thought to provide theoretical ‘genus accumulation curves’, which allow for a comparison of Maastrichtian and Danian diversity at various sampling levels.

With reference to the K-Pg boundary, we assigned each genus to one of five categories: (1) going extinct globally; (2) disappearing in Patagonia but continuing elsewhere; (3) surviving; (4) immigrating; and (5) originating. The reference area for categories 2–5 is Patagonia because geographical restriction to localities may produce strong sampling biases. We categorised genera as immigrants when they occurred in the Danian sections of our study area, had no Cretaceous record from Patagonia, and had been reported outside of Patagonia either before the Danian or within a part of the Danian which is stratigraphically older than our own field records. We categorised genera as originating in Patagonia when they are endemic to this region and first appeared in the Danian, or when a Danian occurrence in our sections is stratigraphically older than records from anywhere else in the world. For each pooled and standardised sample we calculated proportional diversity of the various categories. For the purpose of assigning genera to the five evolutionary/biogeographical categories, geographical and stratigraphical range data were retrieved from the Paleobiology Database (http://paleobiodb.org), Sepkoski’s compendium [19], and numerous monographs on Cretaceous and Palaeogene molluscs. The main sources of information and specific references that were used for the categorisation of taxa are provided in Tables 1 and 2. A full documentation of primary data is presented in Tables S1 and S2.

**Table 1:** Categorisation of molluscs at San Ramòn.

Class	Genus	Range in section	Category	Additional notes and references on records in the	
				Cretaceous	Palaeogene
Bivalvia	*Acesta*	Maa	2		
Gastropoda	*Acteon*	Maa	3		[50]
Bivalvia	*Anomia*	Maa	3		See Table 2
Bivalvia	*Aphrodina*	Danian	3	Valanginian of Argentina[51], [52]	
Bivalvia	*Arcomytilus*	Danian	4		
Bivalvia	*Astarte*	Danian	3	Hauterivian of Argentina [53]	Palaeocene of Argentina [54]
Bivalvia	*Australoneilo*	Maa – Danian	3		
Gastropoda	*Austroaporrhais*	Maa	3		Danian of Patagonia [54], [55]
Bivalvia	*Austrotrigonia*	Maa	1		
Bivalvia	*Bicorbula*	Maa – Danian	3		
Bivalvia	*Brachidontes*	Maa	3		Eocene of Patagonia [56], [57]
Bivalvia	*Camptochlamys*	Maa	1	Casadío et al. [58]	
Bivalvia	*Corbicula*	Maa – Danian	3		
Gastropoda	*Cryptorhytis*	Maa	3		See Table 2
Bivalvia	*Cucullaea*	Maa	3		Danian of Patagonia(unpublished data of authors)
Bivalvia	*Cucullastis*	Danian	4	Late Cretaceous of NewZealand [59]	
Bivalvia	*Cuspidaria*	Danian	4		
Bivalvia	*Eriphyla*	Maa – Danian	3		
Bivalvia	*Etea*	Maa	2		
Gastropoda	*Euspira*	Maa – Danian	3		
Bivalvia	*Glycymeris*	Danian	3	Maastrichtian of Argentina(unpublished data of authorsfrom Sierra Huantraico)	
Gastropoda	*Heteroterma*	Maa – Danian	3		
Bivalvia	*Inoperna*	Maa	1		
Bivalvia	*Lahillia*	Maa	3		Eocene of Patagonia [60]
Bivalvia	*Ledina*	Maa – Danian	3		
Bivalvia	*Leionucula*	Maa – Danian	3		
Bivalvia	*Linearia*	Maa	2		Eocene of USA [61]
Bivalvia	*Lyriochlamys*	Maa	1		
Bivalvia	*Meretrix*	Maa – Danian	3		
Bivalvia	*Mesocallista*	Maa	1		
Bivalvia	*Modiolus*	Maa – Danian	3		
Gastropoda	*Nacella*	Danian	3	See Table 2	
Bivalvia	*Neilo*	Maa	3		Danian [62], Eocene [60] andOligocene [50] of Patagonia
Bivalvia	*Nucula*	Maa	3		Danian of Patagonia [26]
Bivalvia	*Ostrea*	Maa – Danian	3		
Bivalvia	*Pachymya*	Maa	1		
Bivalvia	*Pacificor*	Maa – Danian	3		
Bivalvia	*Pacitrigonia*	Maa	1		
Bivalvia	*Panopea*	Maa – Danian	3		
Gastropoda	*Parasyrinx*	Maa	2		
Bivalvia	*Pholadomya*	Maa	2		
Bivalvia	*Phygraea*	Maa	3		See Table 2
Bivalvia	*Pinna*	Maa – Danian	3		
Gastropoda	*Priscaphander*	Maa	3		See Table 2
Bivalvia	*Protagelus*	Maa	1		
Gastropoda	*Pseudamaura*	Danian	4		[54]
Bivalvia	*Pseudolimea*	Maa	3		See Table 2
Gastropoda	*Pseudotylostoma*	Danian	5		Genus endemic to Patagonia
Bivalvia	*Pteromyrtea*	Maa – Danian	3		
Gastropoda	*Pyropsis*	Maa	2		
Bivalvia	*Rinetrigonia*	Maa	1		
Bivalvia	*Saccella*	Danian	3	Maastrichtian of Argentina(unpublished dataof authors from SierraHuantraico)	
Bivalvia	*Solyma*	Maa	1		
Bivalvia	*Spineilo*	Danian	5		Danian records from Patagoniaare from the lower part of theDanian (this study; foraminiferalzone P1b of [63]), whereas oldestrecords from New Zealand [64]are of late Danian age [26].Palaeocene records from Russiaappear to be late Danian oryounger [65]; see also [66]
Gastropoda	*Struthioptera*	Maa – Danian	3		
Bivalvia	*Tikia*	Maa – Danian	3		
Gastropoda	*Turritella*	Maa – Danian	3		

Alphabetical list of genera (including subgenera) of bivalves and gastropods from the late Maastrichtian and Danian at San Ramón, western Argentina, and their assignment to evolutionary/biogeographical categories.

Evolutionary/biogeographical categories: 1 = going extinct globally in the late Maastrichtian; 2 = disappearing regionally from Patagonia in the late Maastrichtian; 3 = surviving the K-Pg boundary in Patagonia; 4 = immigrating to Patagonia in the Danian; 5 = originating in Patagonia during the Danian. Maa = Maastrichtian.

**Table 2:** Categorisation of molluscs at Bajada del Jagüel and Opaso.

Class	Genus	Range in sections	Category	Additional notes and references on records in the	
				Cretaceous	Palaeogene
Gastropoda	*Acteon*	Maa	3		[50]
Bivalvia	*Ambigostrea*	Maa	1		
Bivalvia	*Amphidonte*	Maa	1		
Bivalvia	*Anomia*	Maa – Danian	3		
Bivalvia	*Aphrodina*	Danian	3	Valanginian ofArgentina [51]	
Bivalvia	*Arcomytilus*	Danian	4		
Bivalvia	*Australoneilo*	Maa – Danian	3		
Bivalvia	*Bicorbula*	Danian	3	See Table 1	
Bivalvia	*Brachidontes*	Maa	3		Eocene of Patagonia [56], [57]
Bivalvia	*Camptochlamys*	Maa	1	[58]	
Bivalvia	*Cosmetodon*	Maa – Danian	3		
Gastropoda	*Cryptorhytis*	Maa – Danian	3		
Bivalvia	*Cucullastis*	Danian	4	Late Cretaceous of NewZealand [59]	
Bivalvia	*Delectopecten*	Danian	4	Early Cretaceous ofGreenland [67]	Danian of Patagonia [62], [68]
Bivalvia	*Disparilia*	Maa – Danian	3		
Bivalvia	*Entolium*	Maa	1		
Bivalvia	*Glycymeris*	Danian	3	Maastrichtian ofArgentina (unpublisheddata of authors fromSierra Huantraico)	
Bivalvia	*Gryphaeostrea*	Danian	3	Aguirre-Urreta [69]	del Río et al. [62], [66]
Gastropoda	*Heteroterma*	Danian	3	See Table 1	
Bivalvia	*Jagolucina*	Danian	5		Thanetian of Australia [70]; Eocene of France [71]
Bivalvia	*Leionucula*	Danian	3	See Table 1	[66]
Bivalvia	*Lima*	Maa	2		
Bivalvia	*Linearia*	Maa	2		Eocene of USA [61]
Bivalvia	*Lyriochlamys*	Maa	1		
Bivalvia	*Mesocallista*	Maa	1		
Bivalvia	*Modiolus*	Maa – Danian	3		
Gastropoda	*Nacella*	Maa – Danian	3		
Bivalvia	*Neilo*	Maa	3		Eocene [60] and Oligocene [50] of Patagonia
Bivalvia	*Nicaniella*	Danian	4		
Bivalvia	*Nuculana*	Maa – Danian	3		
Bivalvia	*Ostrea*	Maa – Danian	3		
Bivalvia	*Pacificor*	Danian	3	See Table 1	
Bivalvia	*Panopea*	Danian	3	See Table 1	
Gastropoda	*Perissodonta*	Danian	5		Danian records from Patagoniaare from the lower part of theDanian (this study;foraminiferal zone P1b of[63]), whereas oldestPalaeocene records from NewZealand [64] are lateDanian [26]
Bivalvia	*Phelopteria*	Maa	1	[72]	
Bivalvia	*Phygraea*	Maa – Danian	3		
Bivalvia	*Pinna*	Danian	3	See Table 1	
Gastropoda	*Priscaphander*	Danian	3	See Table 1	
Bivalvia	*Protagelus*	Maa	1		
Bivalvia	*Pseudolimea*	Maa – Danian	3		
Bivalvia	*Pteromyrtea*	Maa – Danian	3		
Gastropoda	‘*Pyropsis*’	Danian	4		Species belonging to aprobably new genus referred toas ‘*Pyropsis*’ have beendiscussed in [26], [73], [74];oldest records are fromAntarctica
Bivalvia	*Roudairia*	Maa	1		
Bivalvia	*Saccella*	Danian	3	Maastrichtian ofArgentina (unpublisheddata of authors fromSierra Huantraico)	
Bivalvia	*Spineilo*	Danian	5		See Table 1
Bivalvia	*Spondylus*	Maa	2		
Gastropoda	*Struthioptera*	Danian	3	See Table 1	
Gastropoda	*Taioma*	Danian	4	Late Cretaceous ofAntarctica [74]	[74], [75]
Bivalvia	*Tikia*	Maa – Danian	3		
Gastropoda	*Tornatellaea*	Danian	4		Palaeocene of Argentina[54], [75]

Alphabetical list of genera (including subgenera) of bivalves and gastropods from the late Maastrichtian and Danian at Bajada del Jagüel and Opaso, western Argentina, and their assignment to evolutionary/biogeographical categories.

Evolutionary/biogeographical categories: 1 = going extinct globally in the late Maastrichtian; 2 = disappearing regionally from Patagonia in the late Maastrichtian; 3 = surviving the K-Pg boundary in Patagonia; 4 = immigrating to Patagonia in the Danian; 5 = originating in Patagonia during the Danian. Maa = Maastrichtian.

Extinction intensities are expressed as percent extinctions of genera using the data presented in Tables 1 and 2. For comparison with our Patagonian sites, percent global extinctions of molluscan genera were also calculated for three other well-studied K-Pg boundary localities: Brazos River, Texas [20]; Braggs, Alabama [21]; and Stevns Klint, Denmark [22]. These calculations used the primary data provided in the respective publications.

Finally, we compared the relative abundances of the most common Maastrichtian species with their abundances in the Danian and vice versa at both sections. We summed up the total abundances of each species in the Danian and Maastrichtian parts of our respective sections and calculated their proportional abundance in each pooled sample. The proportional abundance of the most common species (those in the upper 90^th^ percentile of all proportional abundances) was then ordered by decreasing proportional abundance and compared with the proportions in the stratigraphically older (for Danian abundances) or younger (for Maastrichtian abundances) parts of the sections. The bivalve *Corbicula*, which is very abundant in the low-diversity brackish-water assemblages, was excluded from this analysis.

All analyses were performed in the R programming environment (www.r-project.org). The field-collected Patagonian specimens are housed at the Departamento de Ciencias Geológicas, Universidad de Buenos Aires, Argentina.

## Results

### Genus-level extinction rates

Intensities of end-Cretaceous extinctions may vary markedly across local, regional, and global scales. In our nearshore section the percentage of global extinctions of benthic molluscan genera is 21.7%. In the offshore section 32.1% of genera became extinct. These values are comparable to those of well-studied Northern Hemisphere boundary sections such as Braggs, Alabama (30.5% extinction of bivalves and gastropods [21]) or Stevns Klint, Denmark (22.5% extinction of bivalves [22]), but markedly higher than at Brazos River, Texas (10.7% extinction of bivalves and gastropods [20]). Local extirpations (60.9% in our nearshore section, 53.6% in the offshore section) are much greater than global extinctions. These higher values are the cumulative result of local extirpations, global extinctions, and sampling effects.

### Maastrichtian and Danian diversity compared

**Figure 1:**
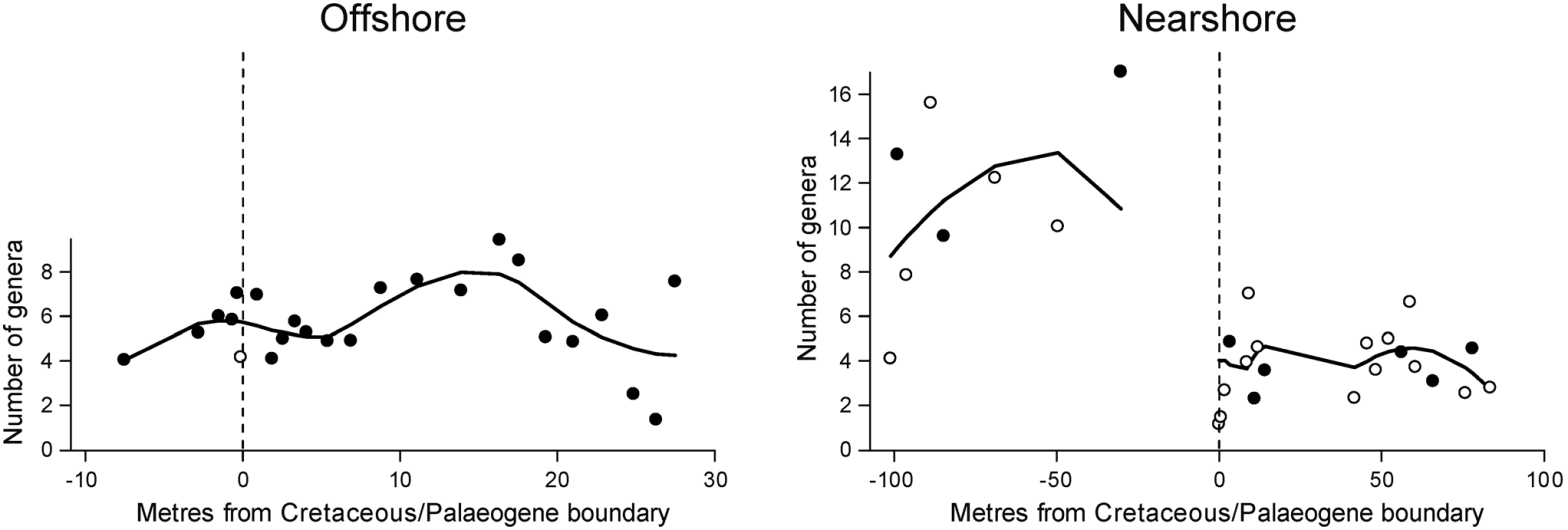
Sample-level diversity of benthic marine molluscs in two Cretaceous-Palaeogene boundary sections in Patagonia. Standardised genus diversity is based on the shareholder quorum subsampling method [17]. Dashed vertical lines indicate position of the Cretaceous-Palaeogene boundary; values on x-axis refer to metres before (Cretaceous, negative values) or after the boundary. Curved solid line is LOESS smoothing with a smoothing parameter of 0.5. Black dots indicate samples from fine-grained sedimentary rocks (siliciclastic mudstones and siltstones), open circles indicate samples from coarse-grained sedimentary rocks (sandstones and conglomerates). Nearshore, the uppermost *ca* 26 metres of the Cretaceous are characterised by low-diversity brackish-water assemblages, which are not included in the figure.

Sample-level diversity in offshore environments fluctuated without a consistent temporal trend (Figure 1). The diversity of even the first quantitative Danian samples is mostly within the range of Maastrichtian assemblages. In contrast, Danian sample-level diversity in nearshore habitats is, on average, distinctly lower than that of pre-extinction assemblages (Figure 1). This pattern is evident in both fine-grained and coarse-grained lithologies and persists until the end of the studied time interval, suggesting contrasting diversity patterns between nearshore and offshore environments. Despite comparable extinction intensities, recovery of sample-level diversity was only achieved offshore.

**Figure 2:**
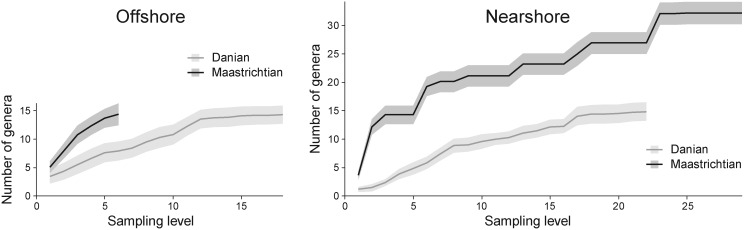
Comparing Cretaceous (Maastrichtian) and Palaeogene (Danian) accumulation curves for the estimated number of molluscan genera. Sampling level refers to consecutively numbered fossiliferous samples, arranged from stratigraphically oldest to youngest, and shows the cumulative number of genera at the respective level (see text for details on pooling and standardising of samples). Grey areas indicate the standard deviations around the means. In both offshore and nearshore habitats total diversity in the Danian is consistently lower than total Maastrichtian diversity at any given sampling level.

Total diversity in the Danian is significantly and consistently lower than total Maastrichtian diversity at the same sampling level (Figure 2). This pattern holds for both nearshore and offshore environments, and points to a distinct difference in diversity structure between Maastrichtian and Danian faunas.

### The contributions of surviving, immigrating, and originating genera

**Figure 3:**
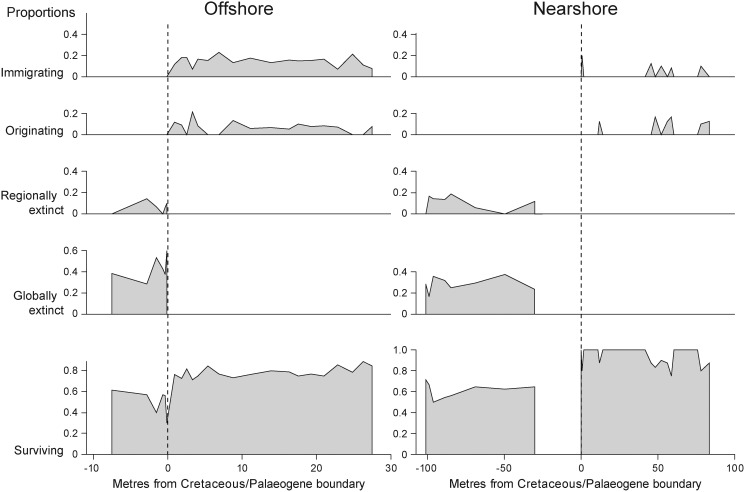
Proportional diversity of molluscan genera in five evolutionary/biogeographical categories across the Cretaceous-Palaeogene boundary. Proportions are shown for an offshore (panels on the left) and a nearshore boundary section (panels on the right) in Patagonia. Dashed vertical lines indicate position of the Cretaceous-Palaeogene boundary; values on x-axis refer to metres before (Cretaceous, negative values) or after the boundary. Nearshore, the uppermost *ca* 26 metres of the Cretaceous are characterised by low-diversity brackish-water assemblages, which are not included in the figure. Post-extinction faunas are dominated by survivors. Immigrating and originating genera play subordinate roles in the composition of Danian samples.

In both environments, the most diverse components of Maastrichtian assemblages are genera that survived the K-Pg boundary event (Figure 3). Immigrating and newly evolving genera play only subordinate roles in the composition of Danian samples. Their occurrence is often sporadic and without obvious trends (Figure 3). The reduced total diversity in the Danian (Figure 2) indicates that immigrants and newly evolved taxa could not compensate for the loss of Maastrichtian diversity at the K-Pg boundary.

### Abundance shifts of dominant species across the K-Pg boundary

**Figure 4:**
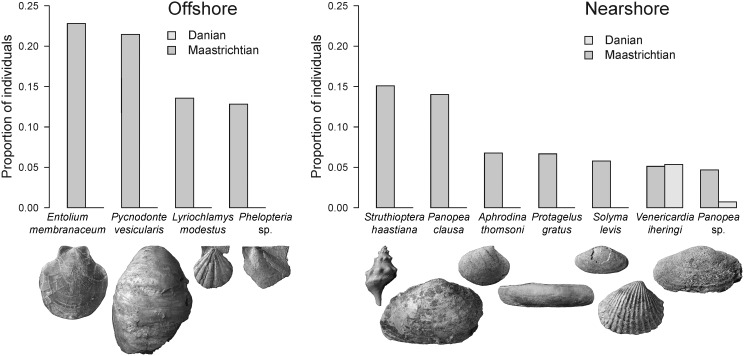
Proportional abundance of most common Maastrichtian molluscs contrasted with their abundances in the Danian. The displayed Maastrichtian species are those in the upper 90^th^ percentile of the pooled samples of that time. Their Danian proportions are plotted alongside. The majority of the dominant Maastrichtian species did not survive into the Danian.

In both environmental settings, the dominant Maastrichtian species became extinct at the K-Pg boundary (Figure 4). In the nearshore environment, this affected the five most abundant Maastrichtian species. Offshore, three of the four most abundant species did not survive the boundary, only the oyster *Pycnodonte vesicularis*, the second most common Maastrichtian species in the offshore environment, survived. However, with only a single specimen found in the Danian, it can be considered as ecologically extinct. It is best classified as a ‘Dead Clade Walking’, a term coined to characterise lineages that survived mass extinctions only to remain marginal or decline in the aftermath [23].

**Figure 5:**
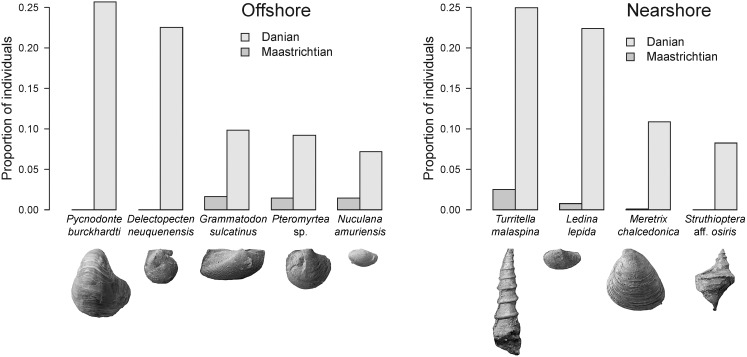
Proportional abundance of most common Danian molluscs contrasted with their abundances in the Maastrichtian. The displayed Danian species are those in the upper 90^th^ percentile of the pooled samples of that time. Their Maastrichtian proportions are plotted alongside. Most of the dominant Danian species are survivors that were rare during the Maastrichtian. The two most abundant Danian offshore species are Patagonian endemics that newly originated in the early Danian.

A second intriguing abundance pattern is seen in species that dominate the Danian assemblages. These were usually rare in the Maastrichtian part of the respective stratigraphic section (Figure 5). The three most abundant nearshore species, collectively making up almost 60% of Danian individuals, are survivors with low abundances during the Maastrichtian. In the offshore environment, three of the five most abundant Danian species are survivors that also changed their relative abundances from rare to common. The two dominant Danian offshore species – the oyster *Pycnodonte burckhardti* and the pectinid *Delectopecten neuquenensis* – are Patagonian endemics that originated in the early Danian.

## Discussion

### Incumbency effects and recovery with minor origination

The persistent dominance of survivors (Figure 3) points to the effectiveness of survival mechanisms [24] and contrasts with recovery models in which a short-term survival phase is followed by diversification owing to origination of new taxa [25]. At any given sampling level, originating and invading genera could not compensate for the loss of genera caused by extinctions (Figure 2). A similar observation was reported for Antarctic molluscs at the K-Pg boundary, where few new genera were observed, and only later in the Danian did the number increase [26].

Delayed recovery is frequently explained by unfavourable environments, either as protracted impact-related effects, or as specific regional effects [27]–[29]. However, local environmental stress fails to explain the Danian *versus* Maastrichtian difference in total diversity in the offshore section where there is no evidence for a shift to more unfavourable environments. Similarly, in the nearshore section the range of sedimentary environments and overall environmental heterogeneity in the Danian and in the Maastrichtian match well [9]. Furthermore, previous work from our working group that used the same raw data from the same nearshore section showed that even Danian assemblages from stable, subtidal environments, characterised by diverse trophic and life habit groups, did not reach Maastrichtian diversity levels [9]. This is exemplified by comparing the Danian *Struthioptera*-*Venericardia* faunal association with the Maastrichtian *Struthioptera*-*Panopea* association of Scasso et al. [9]. Both associations are dominated by infaunal guilds but also have relatively high proportions of epifaunal molluscs. In both associations, trophic resources have been subdivided among suspension-feeders, deposit-feeders, and carnivores, suggesting biologically controlled communities [9]. Yet, the diversity of samples forming the Danian association remains distinctly below the diversity of the Maastrichtian samples. Thus, unfavourable habitats cannot be held responsible for the differences among pre- and post-extinction diversity at either of the two studied sections.

An alternative explanation for the delayed recovery would be low immigration rates caused either by geographic isolation of the basins or by a general shortage in the supply of taxa from outside Patagonia. However, there is no evidence of isolation because the Patagonian marine basins opened to the South Atlantic Ocean to the east, which was a large open ocean with pelagic sedimentation during K-Pg boundary times [9], [10]. Based on general physical oceanographic principles, wind driven surface currents, tidally induced amphidromic circulation, and thermohaline circulation are assumed to have been well developed in the studied areas, all of which would have contributed to the exchange of waters with surrounding regions. The presence in the Neuquén Basin of diverse planktonic foraminifera [11], a plankton group only found in high diversity in marine waters of normal salinity, provides direct evidence for such exchange with open marine waters. Palaeogeographic reconstructions of the marine basins in Patagonia for the Maastrichtian–Palaeocene interval show direct shallow water connections between the basins [9]. Also, with the closely located South American shelf to the north, the Antarctic shelf to the south, and refugia along the margin of the southern-Pacific [26], several potential areas were available as a source for new immigrants. Therefore, geographic isolation and a shortage in the supply of larvae are unlikely scenarios for the delayed recovery in diversity.

We suggest that the numbers of new taxa and of taxa invading from outside Patagonia were low due to the presence of survivors within Patagonia impeding their progress. This phenomenon is known as incumbency or priority effects, in which existing organisms prevent the successful establishment of newly evolving or immigrating species [30], [31]. Resistance to invasions or to the establishment of newly evolved taxa occurs because these ‘would-be usurpers’ are initially less well adapted than established species, and the latter keep incipient populations of new species from taking hold [32], [33]. A closely related concept is that of pre-emptive competition [34], emphasizing the importance of being ‘first in the field’. Incumbency can be expected to play a role when resources are limited and communities are saturated with species [15]. Mass extinctions are generally thought to reduce or remove the incumbency effect [35]. Our analyses, however, suggest that despite significant extinctions, the removal of incumbents did not entail an increase in immigration or origination rates to counterbalance diversity reduction. Rather, the number of survivors seems to have remained sufficiently high to impose constraints on the establishment of immigrants and originating taxa, consistent with incumbency. We consider incumbency as an important mechanism for the delayed recovery in both onshore and offshore environments of Patagonia.

### The extinction of dominants and the rise of rare species

In both studied environments the most common Maastrichtian species became extinct at the K-Pg boundary (Figure 4). This observation appears to be in contrast with ecological studies which suggest that high abundance promotes survival [36]. It can, however, be reconciled with the finding that post-Palaeozoic marine bivalves with high abundances exhibit extinction rates elevated over those of moderately common genera [37]. Also, results of a regional study of North American bivalves showed that abundant taxa were no more likely to survive the K-Pg mass extinction than rare ones [38].

Interestingly, survivors that dominate the Danian assemblages were rare during the Maastrichtian (Figure 5). We suggest that although being resistant to environmental stress, the new dominants were poor competitors before the boundary event. Once they were released from competition by the removal of dominants, the relative competitive ability of survivors increased. This suggests a particular type of incumbency, where numerically subordinate taxa arose to ecological dominance in the aftermath of a major environmental perturbation. Modelling of competitive interactions provides a potential mechanism for such reorganisations of ecosystems [39]: The loss of a competitively superior species releases otherwise unavailable resources, allowing the competitively inferior species to expand its realised niche. Thus, less abundant species may not only provide invasion resistance by their aggregate effects on resource utilization [40], but can be important by gaining dominance after environmental turmoil. Because distinct inversions in abundance across mass extinctions are known from other ecosystems [41] this may indicate a more general macroecological pattern.

The diversity-based conclusions stress the importance of survivors in post-extinction assemblages (Figure 3). This finding is generally confirmed by our abundance-based analyses with one important modification. In the offshore environment, the two top-ranking Danian species are newly evolved bivalves that originated in the early Danian of Patagonia (Figure 5). Their dominance indicates that newly originating post-extinction taxa can have high numbers of individuals even though overall numbers of originating taxa are low. There is a striking taxonomical, morphological, and ecological analogy between these two Danian species and the two most abundant Maastrichtian species in the offshore environment (Figures 4, 5). All four species are epifaunal suspension feeders and it seems that, after its extinction, the Maastrichtian pectinoid *Entolium membranaceum* was replaced by the Danian pectinoid *Delectopecten neuquenensis*, whereas the oyster *Pycnodonte vesicularis* was replaced by the congeneric *Pycnodonte burckhardti*. Although end-Cretaceous extinctions did not remove entire ecological guilds it is plausible that they created enough amount of open ecospace into which taxa with equivalent ecological traits could evolve.

### The pace of recovery in sample-level diversity

The mean sample-level diversity remained unchanged across the K-Pg boundary in the studied Patagonian offshore environments at BJG and Opaso (Figure 1). The early Danian hiatus of *ca* 500 kyr at BJG [11] provides an upper limit for the duration necessary to restore sample-level diversity. The stratigraphically more complete section at Brazos River, east Texas, represents an environment similar to our offshore setting. Sample-level diversity at Brazos started to rebound *ca* 1 myr after the boundary [6] and full local diversity recovery to Cretaceous levels was attained after about 2 myr [42]. Thus, complete recovery of sample-level diversity to pre-extinction values took longer there than in Patagonia. Similarly, another study concluded that the local richness of offshore mollusc assemblages of the U.S. Gulf Coastal Plain recovered to Cretaceous values *ca* 2.7 myr after the event [43]. In conclusion, the time necessary to recover to pre-extinction diversity in mollusc-dominated offshore ecosystems varies and may take less than *ca* 0.5 myr according to our results from Patagonia and up to *ca* 2.7 myr according to data from the U.S. Gulf Coastal Plain. In contrast to offshore settings, Danian sample-level diversity did not recover to Maastrichtian levels in the Patagonian nearshore environments. We have shown from the data that there is a prolonged period of incomplete recovery in nearshore environments, although a lack of more detailed chronostratigraphic information prevents us from quantifying its duration more precisely.

### Divergence of Maastrichtian and Danian diversity structure

Accumulation of total Danian diversity slowed down over time (Figure 2), seemingly compatible with a model of diversity-dependent niche-filling and a gradual approach to carrying capacity. These curves, however, should not be taken as accurate reflections of the rate at which post-extinction diversity builds up because they build on the assumption that (1) all genera found in any sample in the Danian are also present in all younger Danian samples, and (2) any genera not found in older samples represent true absences.

Geological time is critical for our interpretation that Figure 2 indicates systematic differences between Maastrichtian and Danian biodiversity. For example, the underlying (re-)population dynamics may be the same for each interval, but an apparent difference could be generated if Maastrichtian strata represented a much longer time span than Danian strata. Because the opposite is true in the offshore section (Danian samples encompass about twice as much time as Maastrichtian samples) any potential bias would operate only in the reverse direction. Interval lengths are less clear in the nearshore section but we assume roughly equal amounts of time (see above).

The reduced total diversity in the Danian and the longer-term stability of sample-level diversity across the boundary in offshore habitats are no contradiction. Jointly these patterns imply that, on average, Danian offshore assemblages retained, or rapidly regained, their Maastrichtian sample-level diversity despite being recruited from an overall smaller pool of Danian taxa. This indicates a shift in the partitioning of sample-level diversity and total diversity.

Erwin [44] pointed out that positive feedbacks may increase carrying capacity during biotic recovery. An example of a positive feedback in mollusc-dominated ecosystems may be the Cenozoic radiation of predatory gastropods and the resulting escalatory trends ([43] and references therein). If such effects were present in our area, they must have occurred subsequent to the studied time interval.

### Nearshore–offshore differentials in recovery

In contrast to the offshore environment, the reduction in nearshore Danian sample-level diversity was pronounced and lasted over the entire studied interval. Differential evolutionary dynamics along onshore–offshore gradients – well-known in post-Palaeozoic benthic marine invertebrates, with preferential origination occurring onshore [45], [46] – is inadequate to account for the observed difference in our study. Not only would this mechanism favour the recovery of onshore diversity – which is just the opposite of the pattern we observe – but originations in general played only a minor role during recovery in our study areas. The nearshore–offshore difference might have arisen from unpredictable, stochastic processes, consistent with the contingent recovery model [47] which emphasises the importance of chance in the recovery of marine plankton from the end-Cretaceous extinction. A more mechanistic explanation focuses on the role of survivors as the most diverse group. We suggest that the recolonisation by survivors from nearby localities may have been facilitated in the spatially and temporally more homogeneous and continuous offshore environment. Under such conditions we expect that populations are well connected with each other. In ecology, high levels of connectivity within metapopulations are often considered to be beneficial for recovery after local depletion or extirpation [48]. In contrast, species inhabiting the more variable nearshore environments tend to have more patchy distributions. Their supposedly lower connectivity among populations may have hampered the recolonisation by survivors in nearshore settings.

## Conclusions

We analysed so far underrepresented K-Pg boundary sections from the Southern Hemisphere, which contribute to a more complete understanding of recovery processes. Overall, our results corroborate and extend earlier work finding interregional and environmental variation in recovery dynamics in benthic [3], [7] and planktonic marine systems [28], [29]. Diversity of the post-extinction interval was controlled by taxa that survived the extinction event in Patagonia. We emphasise the importance of biotic interactions and conclude that survivors had negative effects on the potential origination of new taxa and the invasion of exotic genera through incumbency. Furthermore, survivors constituted the pool of taxa from which the majority of new dominants were recruited after the most abundant Maastrichtian species went extinct at the boundary. Such distinct shifts in the abundances of survivors are a feature these benthic marine ecosystems share with terrestrial plant communities across mass extinction boundaries [49]. Restoration of sample-level diversity of mollusc assemblages can be rapid (< *ca* 500 kyr in offshore habitats). In contrast, total Danian diversity remained below that of the Maastrichtian, despite a marked Palaeogene increase in the global diversity of gastropods and bivalves [8].

## Supporting Information

Table S1 Primary data of San Ramón.
(XLS)

Table S2 Primary data of Bajada del Jagüel and Opaso.
(XLS)
